# Triethyl­ammonium (2*R*,3*R*)-2,3-bis­(benzo­yloxy)-3-carb­oxy­propano­ate

**DOI:** 10.1107/S1600536812004308

**Published:** 2012-02-10

**Authors:** Shang-Ju Li, Rong-Jia Zhang, Guang-Feng Hou, Guang-Ming Li, Peng-Fei Yan

**Affiliations:** a Key Laboratory of Functional Inorganic Material Chemistry (HLJU), Ministry of Education, and School of Chemistry and Materials Science, Heilongjiang University, Harbin 150080, People’s Republic of China

## Abstract

In the anion of the title salt, C_6_H_16_N^+^·C_18_H_13_O_8_^−^, one of the carboxyl groups is deprotonated. Its O atoms are involved in inter­molecular hydrogen bonding with the carboxyl group of an adjacent anion and the amino group of an adjacent cation. The two benzoyloxy rings are oriented with respect to each other at a dihedral angle of 79.46 (6)°.

## Related literature

For background to tartaric acid derivatives, see: Kassai *et al.* (2000 ▶); Tan *et al.* (2006 ▶).
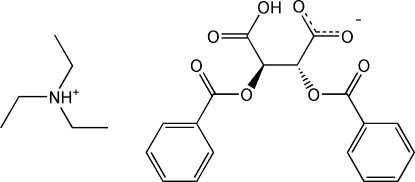


## Experimental

### 

#### Crystal data

C_6_H_16_N^+^·C_18_H_13_O_8_^−^
*M* *_r_* = 459.48
Orthorhombic, 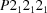

*a* = 11.2148 (12) Å
*b* = 12.9835 (13) Å
*c* = 15.9499 (17) Å
*V* = 2322.4 (4) Å^3^
*Z* = 4
Mo *K*α radiation
μ = 0.10 mm^−1^
*T* = 293 K
0.20 × 0.20 × 0.18 mm

#### Data collection

Rigaku R-AXIS RAPID diffractometer
17272 measured reflections
3242 independent reflections
2336 reflections with *I* > 2σ(*I*)
*R*_int_ = 0.067

#### Refinement

*R*[*F* ^2^ > 2σ(*F* ^2^)] = 0.042
*wR*(*F* ^2^) = 0.095
*S* = 1.02
3242 reflections
309 parameters
H atoms treated by a mixture of independent and constrained refinement
Δρ_max_ = 0.20 e Å^−3^
Δρ_min_ = −0.21 e Å^−3^

### 

Data collection: *RAPID-AUTO* (Rigaku, 1998 ▶); cell refinement: *RAPID-AUTO*; data reduction: *CrystalClear* (Rigaku/MSC, 2002 ▶); program(s) used to solve structure: *SHELXTL* (Sheldrick, 2008 ▶); program(s) used to refine structure: *SHELXTL*; molecular graphics: *SHELXTL*; software used to prepare material for publication: *SHELXTL*.

## Supplementary Material

Crystal structure: contains datablock(s) I, global. DOI: 10.1107/S1600536812004308/xu5427sup1.cif

Structure factors: contains datablock(s) I. DOI: 10.1107/S1600536812004308/xu5427Isup2.hkl

Supplementary material file. DOI: 10.1107/S1600536812004308/xu5427Isup3.cml

Additional supplementary materials:  crystallographic information; 3D view; checkCIF report

## Figures and Tables

**Table 1:** Hydrogen-bond geometry (Å, °)

*D*—H⋯*A*	*D*—H	H⋯*A*	*D*⋯*A*	*D*—H⋯*A*
N1—H101⋯O4^i^	0.95 (3)	1.82 (3)	2.770 (3)	177 (3)
O5—H51⋯O3^ii^	0.93 (4)	1.60 (3)	2.525 (2)	171 (3)

Symmetry codes: (i) 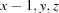; (ii) 
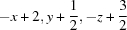
.

**Fig. 1.**
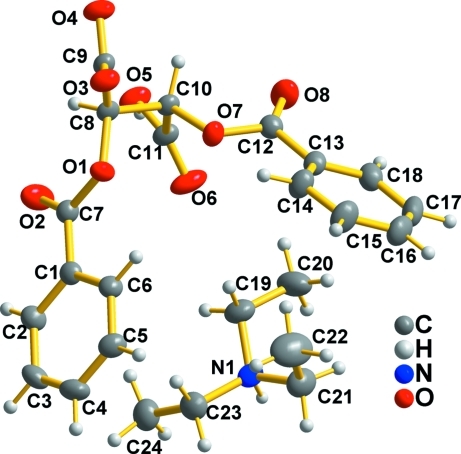
The molecular structure of the title compound, showing 50% probability displacement ellipsoids.

**Fig. 2.**
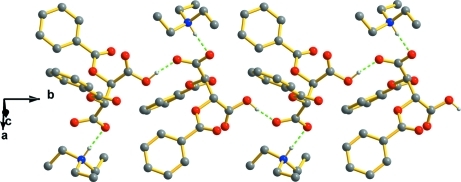
A partial packing view, showing the hydrogen bonding chain structure along [010].

## supplementary crystallographic information

### Comment

Optically active tartaric acid and its acyl derivatives (*e.g.* diacetyl, dibenzoyl, di-*p*-toluyl) continue to be widely used in the resolution of racemates. Their resolving power is connected with the ability to form diastereomeric salts with racemic amines and hydrazides. (2R,3R)-O,O'-Dibenzoyl-tartaric acid (DBTA) has been known as a chiral selector of enantiomers, such as ephedrine, chiral alcohols and N-methylamphetamine. It was also observed that DBTA can form complexes with some alcohols and aminos; the complex forming properties of DBTA have been investigated (Kassai *et al.*, 2000; Tan *et al.*, 2006).

The asymmertric unit of title compound contains one (2R,3R)-O,O'-dibenzoyl-tartrate anion and one triethylamine cation. In the anion, the two benzene rings make a dihedral angle of 79.46 (6)° (Fig. 1). In the crystal packing, intermoleculear O—H···O and N—H···O hydrogen bonds link these cations and anions into chain structures along [010] (Fig. 2, Table 1).

### Experimental

(2R,3R)-O,O'-Dibenzoyl-tartaric acid was a commercially available compound and used as received without further purification. (2R,3R)-O,O'-Dibenzoyl-tartaric acid (0.358 g, 1.0 mmol) and triethylamine (1 mL, 2 mol/L) were dissolved in methanol (8 mL), and the solution was stirring for 10 min at room temperature. And then, the solution was stood at room temperature for a few days, colorless block crystals of title compound were obtained (yield 27%).

### Refinement

O-bound and N-bound H atoms were located in a difference Fourier map and refined freely. H atoms bound to C atoms were placed in calculated positions and treated as riding on their parent atoms with C—H = 0.93–0.98 Å, U_iso_(H) = 1.5U_eq_(C) for methyl H atoms and 1.2U_eq_(C) for the opthers. As no significant anomalous scatterings, Friedel pairs were merged, the enantiomer has been assigned by reference to an unchanging chiral centre in the synthetic procedure.

### Crystal data

**Table:**

C_6_H_16_N^+^·C_18_H_13_O_8_^−^	*F*(000) = 976
*M**_r_* = 459.48	*D*_x_ = 1.314 Mg m^−^^3^
Orthorhombic, *P*2_1_2_1_2_1_	Mo *K*α radiation, λ = 0.71073 Å
Hall symbol: P 2ac 2ab	Cell parameters from 17618 reflections
*a* = 11.2148 (12) Å	θ = 2.4–28.3°
*b* = 12.9835 (13) Å	µ = 0.10 mm^−^^1^
*c* = 15.9499 (17) Å	*T* = 293 K
*V* = 2322.4 (4) Å^3^	Block, colorless
*Z* = 4	0.20 × 0.20 × 0.18 mm

### Data collection

**Table:**

Rigaku R-AXIS RAPID diffractometer	2336 reflections with *I* > 2σ(*I*)
Radiation source: fine-focus sealed tube	*R*_int_ = 0.067
Graphite monochromator	θ_max_ = 28.3°, θ_min_ = 2.4°
ω scan	*h* = −14→14
17272 measured reflections	*k* = −17→17
3242 independent reflections	*l* = −21→18

### Refinement

**Table:**

Refinement on *F*^2^	Primary atom site location: structure-invariant direct methods
Least-squares matrix: full	Secondary atom site location: difference Fourier map
*R*[*F*^2^ > 2σ(*F*^2^)] = 0.042	Hydrogen site location: inferred from neighbouring sites
*wR*(*F*^2^) = 0.095	H atoms treated by a mixture of independent and constrained refinement
*S* = 1.02	*w* = 1/[σ^2^(*F*_o_^2^) + (0.0431*P*)^2^ + 0.0369*P*] where *P* = (*F*_o_^2^ + 2*F*_c_^2^)/3
3242 reflections	(Δ/σ)_max_ < 0.001
309 parameters	Δρ_max_ = 0.20 e Å^−^^3^
0 restraints	Δρ_min_ = −0.21 e Å^−^^3^

### Special details

**Table:**

Geometry. All esds (except the esd in the dihedral angle between two l.s. planes) are estimated using the full covariance matrix. The cell esds are taken into account individually in the estimation of esds in distances, angles and torsion angles; correlations between esds in cell parameters are only used when they are defined by crystal symmetry. An approximate (isotropic) treatment of cell esds is used for estimating esds involving l.s. planes.
Refinement. merg 4 Refinement of F^2^ against ALL reflections. The weighted R-factor wR and goodness of fit S are based on F^2^, conventional R-factors R are based on F, with F set to zero for negative F^2^. The threshold expression of F^2^ > 2sigma(F^2^) is used only for calculating R-factors(gt) etc. and is not relevant to the choice of reflections for refinement. R-factors based on F^2^ are statistically about twice as large as those based on F, and R- factors based on ALL data will be even larger.

### Fractional atomic coordinates and isotropic or equivalent isotropic displacement parameters (Å^2^)

**Table:**

	*x*	*y*	*z*	*U*_iso_*/*U*_eq_	
C1	0.8315 (2)	0.31475 (18)	0.91650 (17)	0.0256 (6)	
C2	0.7651 (2)	0.3345 (2)	0.98812 (17)	0.0318 (6)	
H2	0.7737	0.3971	1.0157	0.038*	
C3	0.6859 (3)	0.2615 (2)	1.01896 (18)	0.0355 (7)	
H3	0.6412	0.2752	1.0668	0.043*	
C4	0.6740 (2)	0.1687 (2)	0.97802 (19)	0.0366 (7)	
H4	0.6215	0.1195	0.9988	0.044*	
C5	0.7395 (2)	0.14762 (19)	0.90625 (18)	0.0329 (6)	
H5	0.7306	0.0848	0.8790	0.039*	
C6	0.8185 (2)	0.22080 (18)	0.87521 (17)	0.0272 (6)	
H6	0.8625	0.2071	0.8271	0.033*	
C7	0.9139 (2)	0.3955 (2)	0.88549 (16)	0.0267 (6)	
C8	1.0565 (2)	0.43421 (16)	0.77853 (17)	0.0239 (5)	
H8	1.0692	0.4933	0.8157	0.029*	
C9	1.1775 (2)	0.38534 (17)	0.75872 (16)	0.0241 (5)	
C10	0.9965 (2)	0.47203 (17)	0.69826 (16)	0.0237 (5)	
H10	1.0568	0.5027	0.6614	0.028*	
C11	0.8990 (2)	0.55163 (17)	0.71601 (18)	0.0273 (6)	
C12	0.9025 (2)	0.3985 (2)	0.58089 (18)	0.0283 (6)	
C13	0.8401 (2)	0.30655 (18)	0.54713 (17)	0.0284 (6)	
C14	0.8626 (2)	0.2078 (2)	0.5773 (2)	0.0362 (7)	
H14	0.9170	0.1978	0.6205	0.043*	
C15	0.8031 (3)	0.1247 (2)	0.5423 (2)	0.0437 (8)	
H15	0.8191	0.0584	0.5614	0.052*	
C16	0.7203 (3)	0.1396 (2)	0.4795 (2)	0.0432 (8)	
H16	0.6806	0.0834	0.4566	0.052*	
C17	0.6964 (3)	0.2369 (2)	0.45069 (19)	0.0411 (7)	
H17	0.6394	0.2467	0.4091	0.049*	
C18	0.7572 (2)	0.3209 (2)	0.48357 (18)	0.0363 (7)	
H18	0.7423	0.3867	0.4630	0.044*	
C19	0.5614 (2)	0.4077 (2)	0.7214 (2)	0.0410 (7)	
H19A	0.6369	0.3714	0.7220	0.049*	
H19B	0.5653	0.4619	0.7631	0.049*	
C20	0.5432 (3)	0.4554 (3)	0.6361 (2)	0.0549 (9)	
H20A	0.4659	0.4871	0.6337	0.082*	
H20B	0.5486	0.4030	0.5938	0.082*	
H20C	0.6034	0.5066	0.6265	0.082*	
C21	0.4518 (3)	0.2449 (2)	0.6854 (2)	0.0455 (8)	
H21A	0.4350	0.2709	0.6296	0.055*	
H21B	0.3841	0.2037	0.7029	0.055*	
C22	0.5604 (3)	0.1767 (2)	0.6811 (3)	0.0685 (11)	
H22A	0.5752	0.1471	0.7353	0.103*	
H22B	0.6281	0.2168	0.6641	0.103*	
H22C	0.5470	0.1226	0.6411	0.103*	
C23	0.4718 (3)	0.2984 (2)	0.83353 (18)	0.0391 (7)	
H23A	0.5499	0.2684	0.8429	0.047*	
H23B	0.4129	0.2447	0.8424	0.047*	
C24	0.4518 (3)	0.3828 (2)	0.89667 (19)	0.0479 (8)	
H24A	0.3797	0.4191	0.8832	0.072*	
H24B	0.5179	0.4298	0.8954	0.072*	
H24C	0.4451	0.3534	0.9517	0.072*	
N1	0.46413 (19)	0.33463 (16)	0.74420 (14)	0.0308 (5)	
H101	0.391 (3)	0.372 (2)	0.7409 (18)	0.045 (8)*	
O1	0.98006 (14)	0.36173 (11)	0.82011 (11)	0.0255 (4)	
O2	0.92394 (19)	0.48083 (14)	0.91440 (14)	0.0451 (6)	
O3	1.19370 (14)	0.29069 (12)	0.76840 (12)	0.0313 (4)	
O4	1.25517 (14)	0.44777 (12)	0.73477 (13)	0.0334 (4)	
O5	0.94968 (15)	0.64074 (12)	0.73381 (13)	0.0339 (4)	
H51	0.891 (3)	0.691 (2)	0.735 (2)	0.076 (11)*	
O6	0.79444 (15)	0.53471 (14)	0.71332 (15)	0.0447 (6)	
O7	0.94704 (15)	0.38233 (11)	0.65836 (11)	0.0270 (4)	
O8	0.91205 (18)	0.47972 (14)	0.54453 (13)	0.0425 (5)	

### Atomic displacement parameters (Å^2^)

**Table:**

	*U*^11^	*U*^22^	*U*^33^	*U*^12^	*U*^13^	*U*^23^
C1	0.0253 (11)	0.0246 (13)	0.0270 (15)	0.0042 (10)	−0.0020 (11)	0.0015 (11)
C2	0.0355 (14)	0.0283 (13)	0.0315 (16)	0.0038 (12)	0.0048 (13)	−0.0028 (12)
C3	0.0363 (14)	0.0421 (16)	0.0283 (17)	0.0065 (13)	0.0100 (13)	0.0046 (13)
C4	0.0322 (14)	0.0350 (15)	0.0426 (18)	−0.0020 (13)	0.0084 (13)	0.0103 (14)
C5	0.0306 (13)	0.0243 (13)	0.0437 (18)	0.0006 (11)	0.0014 (13)	−0.0002 (12)
C6	0.0255 (12)	0.0265 (12)	0.0295 (15)	0.0030 (11)	0.0031 (11)	−0.0018 (11)
C7	0.0268 (13)	0.0246 (13)	0.0285 (15)	0.0013 (11)	−0.0008 (11)	−0.0025 (11)
C8	0.0263 (11)	0.0161 (10)	0.0294 (14)	−0.0049 (10)	0.0002 (12)	−0.0022 (10)
C9	0.0246 (12)	0.0202 (11)	0.0274 (15)	−0.0021 (10)	−0.0035 (11)	0.0014 (10)
C10	0.0243 (12)	0.0172 (10)	0.0296 (15)	−0.0023 (10)	−0.0013 (11)	0.0000 (10)
C11	0.0249 (12)	0.0187 (11)	0.0383 (17)	−0.0009 (10)	−0.0017 (12)	0.0025 (11)
C12	0.0277 (12)	0.0270 (13)	0.0303 (16)	0.0037 (11)	−0.0007 (12)	0.0006 (12)
C13	0.0257 (12)	0.0303 (14)	0.0294 (16)	0.0040 (11)	−0.0025 (12)	−0.0026 (12)
C14	0.0358 (15)	0.0296 (14)	0.0431 (19)	0.0004 (12)	−0.0113 (13)	−0.0021 (13)
C15	0.0478 (17)	0.0294 (14)	0.054 (2)	−0.0012 (14)	−0.0105 (16)	−0.0101 (15)
C16	0.0404 (16)	0.0432 (17)	0.046 (2)	−0.0043 (14)	−0.0063 (15)	−0.0155 (15)
C17	0.0354 (15)	0.0553 (19)	0.0327 (18)	0.0036 (14)	−0.0099 (14)	−0.0102 (15)
C18	0.0354 (14)	0.0421 (16)	0.0314 (17)	0.0063 (13)	−0.0029 (13)	−0.0019 (13)
C19	0.0281 (13)	0.0426 (15)	0.052 (2)	−0.0040 (12)	−0.0004 (15)	0.0036 (15)
C20	0.0392 (17)	0.069 (2)	0.057 (2)	−0.0049 (17)	0.0068 (16)	0.0195 (18)
C21	0.0456 (17)	0.0407 (16)	0.050 (2)	−0.0039 (15)	0.0022 (16)	−0.0131 (15)
C22	0.060 (2)	0.0522 (19)	0.093 (3)	0.0086 (18)	0.020 (2)	−0.022 (2)
C23	0.0418 (16)	0.0371 (15)	0.0383 (18)	0.0030 (13)	−0.0024 (14)	0.0056 (14)
C24	0.0506 (18)	0.0524 (18)	0.0405 (19)	0.0004 (16)	−0.0060 (16)	−0.0023 (15)
N1	0.0242 (10)	0.0305 (11)	0.0378 (15)	0.0030 (9)	−0.0022 (10)	−0.0008 (10)
O1	0.0282 (8)	0.0192 (8)	0.0291 (10)	−0.0022 (7)	0.0072 (8)	−0.0021 (7)
O2	0.0550 (13)	0.0276 (10)	0.0527 (14)	−0.0101 (9)	0.0192 (11)	−0.0143 (10)
O3	0.0269 (9)	0.0192 (8)	0.0477 (12)	0.0003 (7)	0.0017 (9)	0.0023 (9)
O4	0.0238 (8)	0.0243 (8)	0.0522 (13)	−0.0013 (8)	0.0029 (9)	0.0083 (9)
O5	0.0264 (8)	0.0164 (8)	0.0590 (13)	0.0011 (7)	−0.0004 (9)	−0.0024 (9)
O6	0.0221 (9)	0.0313 (9)	0.0805 (17)	−0.0026 (8)	0.0022 (10)	−0.0082 (11)
O7	0.0337 (9)	0.0179 (8)	0.0292 (11)	−0.0028 (7)	−0.0062 (8)	−0.0009 (7)
O8	0.0509 (12)	0.0318 (10)	0.0448 (13)	−0.0052 (9)	−0.0112 (11)	0.0133 (10)

### Geometric parameters (Å, º)

**Table:**

C1—C2	1.388 (4)	C14—H14	0.9300
C1—C6	1.394 (3)	C15—C16	1.380 (4)
C1—C7	1.482 (3)	C15—H15	0.9300
C2—C3	1.389 (4)	C16—C17	1.371 (4)
C2—H2	0.9300	C16—H16	0.9300
C3—C4	1.377 (4)	C17—C18	1.389 (4)
C3—H3	0.9300	C17—H17	0.9300
C4—C5	1.387 (4)	C18—H18	0.9300
C4—H4	0.9300	C19—N1	1.491 (3)
C5—C6	1.390 (4)	C19—C20	1.508 (4)
C5—H5	0.9300	C19—H19A	0.9700
C6—H6	0.9300	C19—H19B	0.9700
C7—O2	1.205 (3)	C20—H20A	0.9600
C7—O1	1.353 (3)	C20—H20B	0.9600
C8—O1	1.435 (3)	C20—H20C	0.9600
C8—C10	1.527 (3)	C21—N1	1.502 (3)
C8—C9	1.532 (3)	C21—C22	1.507 (4)
C8—H8	0.9800	C21—H21A	0.9700
C9—O4	1.249 (3)	C21—H21B	0.9700
C9—O3	1.252 (3)	C22—H22A	0.9600
C10—O7	1.439 (3)	C22—H22B	0.9600
C10—C11	1.531 (3)	C22—H22C	0.9600
C10—H10	0.9800	C23—N1	1.503 (4)
C11—O6	1.194 (3)	C23—C24	1.505 (4)
C11—O5	1.320 (3)	C23—H23A	0.9700
C12—O8	1.208 (3)	C23—H23B	0.9700
C12—O7	1.349 (3)	C24—H24A	0.9600
C12—C13	1.485 (3)	C24—H24B	0.9600
C13—C18	1.388 (4)	C24—H24C	0.9600
C13—C14	1.392 (4)	N1—H101	0.95 (3)
C14—C15	1.386 (4)	O5—H51	0.93 (4)
			
C2—C1—C6	119.6 (2)	C15—C16—H16	119.8
C2—C1—C7	118.6 (2)	C16—C17—C18	120.1 (3)
C6—C1—C7	121.8 (2)	C16—C17—H17	120.0
C1—C2—C3	120.6 (3)	C18—C17—H17	120.0
C1—C2—H2	119.7	C13—C18—C17	119.9 (3)
C3—C2—H2	119.7	C13—C18—H18	120.0
C4—C3—C2	119.4 (3)	C17—C18—H18	120.0
C4—C3—H3	120.3	N1—C19—C20	112.5 (2)
C2—C3—H3	120.3	N1—C19—H19A	109.1
C3—C4—C5	120.9 (2)	C20—C19—H19A	109.1
C3—C4—H4	119.6	N1—C19—H19B	109.1
C5—C4—H4	119.6	C20—C19—H19B	109.1
C4—C5—C6	119.8 (2)	H19A—C19—H19B	107.8
C4—C5—H5	120.1	C19—C20—H20A	109.5
C6—C5—H5	120.1	C19—C20—H20B	109.5
C5—C6—C1	119.8 (2)	H20A—C20—H20B	109.5
C5—C6—H6	120.1	C19—C20—H20C	109.5
C1—C6—H6	120.1	H20A—C20—H20C	109.5
O2—C7—O1	122.8 (2)	H20B—C20—H20C	109.5
O2—C7—C1	125.5 (2)	N1—C21—C22	114.2 (3)
O1—C7—C1	111.7 (2)	N1—C21—H21A	108.7
O1—C8—C10	109.60 (19)	C22—C21—H21A	108.7
O1—C8—C9	110.67 (17)	N1—C21—H21B	108.7
C10—C8—C9	110.5 (2)	C22—C21—H21B	108.7
O1—C8—H8	108.7	H21A—C21—H21B	107.6
C10—C8—H8	108.7	C21—C22—H22A	109.5
C9—C8—H8	108.7	C21—C22—H22B	109.5
O4—C9—O3	125.0 (2)	H22A—C22—H22B	109.5
O4—C9—C8	114.34 (19)	C21—C22—H22C	109.5
O3—C9—C8	120.6 (2)	H22A—C22—H22C	109.5
O7—C10—C8	106.28 (17)	H22B—C22—H22C	109.5
O7—C10—C11	110.67 (19)	N1—C23—C24	113.4 (2)
C8—C10—C11	112.1 (2)	N1—C23—H23A	108.9
O7—C10—H10	109.2	C24—C23—H23A	108.9
C8—C10—H10	109.2	N1—C23—H23B	108.9
C11—C10—H10	109.2	C24—C23—H23B	108.9
O6—C11—O5	126.3 (2)	H23A—C23—H23B	107.7
O6—C11—C10	124.8 (2)	C23—C24—H24A	109.5
O5—C11—C10	108.9 (2)	C23—C24—H24B	109.5
O8—C12—O7	122.9 (2)	H24A—C24—H24B	109.5
O8—C12—C13	124.7 (2)	C23—C24—H24C	109.5
O7—C12—C13	112.4 (2)	H24A—C24—H24C	109.5
C18—C13—C14	119.8 (2)	H24B—C24—H24C	109.5
C18—C13—C12	118.2 (2)	C19—N1—C21	114.1 (2)
C14—C13—C12	122.0 (2)	C19—N1—C23	112.9 (2)
C15—C14—C13	119.4 (3)	C21—N1—C23	110.8 (2)
C15—C14—H14	120.3	C19—N1—H101	107.3 (17)
C13—C14—H14	120.3	C21—N1—H101	106.1 (17)
C16—C15—C14	120.4 (3)	C23—N1—H101	105.0 (18)
C16—C15—H15	119.8	C7—O1—C8	118.11 (18)
C14—C15—H15	119.8	C11—O5—H51	108 (2)
C17—C16—C15	120.3 (3)	C12—O7—C10	114.96 (18)
C17—C16—H16	119.8		

### Hydrogen-bond geometry (Å, º)

**Table:**

*D*—H···*A*	*D*—H	H···*A*	*D*···*A*	*D*—H···*A*
N1—H101···O4^i^	0.95 (3)	1.82 (3)	2.770 (3)	177 (3)
O5—H51···O3^ii^	0.93 (4)	1.60 (3)	2.525 (2)	171 (3)

Symmetry codes: (i) *x*−1, *y*, *z*; (ii) −*x*+2, *y*+1/2, −*z*+3/2.
